# A New Inner Fabrication Method of Internal Cavity in Metal under Compound Acoustic Fields

**DOI:** 10.3390/mi14040719

**Published:** 2023-03-24

**Authors:** Zheng Zhang, Liqun Wu, Yaxing Wang, Ze’en Wang, Guanwu Wu, Yajing Wang, Hongcheng Wang

**Affiliations:** School of Mechanical Engineering, Hangzhou Dianzi University, Hangzhou 310018, China; 212010124@hdu.edu.cn (Z.Z.); 202010053@hdu.edu.cn (Z.W.); guanwu_cc@hdu.edu.cn (G.W.); 202010139@hdu.edu.cn (Y.W.);

**Keywords:** metal, inner micro-cavity, compound acoustic fields, cavitation and levitation, internal processing method

## Abstract

In order to realize direct manufacturing of cavity inside metal without assembly, this paper investigates the mechanism of cavity manufacturing inside metals under compound acoustic fields. First, a local acoustic cavitations model is established to study the single bubble generation at fixed point in Ga-In metal droplets, which has a low melting point. Second, cavitation-levitation acoustic composite fields are integrated with the experimental system for simulation and experimentation. Through COMSOL simulation and experimentation, this paper expounds the manufacturing mechanism of metal internal cavity under acoustic composite fields. The key problem is to control the duration of the cavitation bubble by controlling the driving acoustic pressure’s frequency and the magnitude of ambient acoustic pressure. Under the condition of composite acoustic fields, this method realizes the direct fabrication of cavity inside Ga-In alloy for the first time.

## 1. Introduction

In 2004, Professor Hanmin Shi proposed ‘the internal processing method [1]’, which brings the processing energy to the interior of the material, targeting specific positions to fabricate desired structures or physical and chemical properties. Currently, internal processing technology is widely studied. Among these studies, femtosecond laser processing has achieved significant research results and application value in the internal processing of transparent materials, such as silicon and polymer [2,3,4,5]. Compared to traditional processing methods, the development of internal processing technology is expected to solve the problems of complex processing technology, high energy consumption, and difficulty in manufacturing complex microstructures. However, the flaw of laser internal processing technology is also evident, which is the inability to process the interior of non-transparent materials, such as non-ferrous metals. Based on this, this paper proposes the application of ultrasonic energy to the interior of metal to realize direct processing inside of metal, which is expected to solve the technical problem that femtosecond laser processing cannot process the interior of metal.

To achieve ultrasonic internal machining, two challenges need to be addressed: controlling the position and the size of cavitation bubbles. In 1949, Rayleigh proposed the bubble dynamics equation for analyzing the cavitation flow in a fluid field with a relatively small number of cavitation bubbles [6]. Subsequently, Noltingk, Neppiras, and Poritsky modified Rayleigh’s equation to account for the dynamics of bubbles in a viscous fluid [7]. This signifies the establishment of the classical equation that describes the dynamic behavior of bubbles in fluid. Based on the solution and correction of this equation, researchers have conducted a lot of research on ultrasonic cavitation behavior. In 2002, Yutaka Abe et al. studied the precise control of bubble position in a standing wave field. Through experiments, it was confirmed that ultrasonic standing waves can accurately control the position of bubbles in the liquid [8]. In 2015, Sheng Zhuang-Zhi studied the position of ultrasonic cavitation bubble in liquid and the direction of motion under the action of the primary Bjerknes force [9]. In 2018, Zang et al. utilized acoustic levitation to open and close the shell of liquid particles and proposed a new technology for generating cavitation bubbles from droplets. When the cavity volume reached a critical point, the acoustic resonance induced buckling instability, leading to a sharp increase in the volume of the cavitation bubble induced by the acoustic standing wave field. This resulted in the dramatic expansion of the cavity and its rapid closure, forming the cavitation bubble [10,11,12,13,14,15,16,17,18]. The buckling instability of the liquid film under acoustic levitation provides a method for converting droplets into large cavitation bubbles. In 2020, Chen Shu et al. studied the characteristics of thin metal liquid cavitation bubbles, but the number, size, and location of cavitation bubbles were uncontrollable [19,20]. Research on cavitation bubbles in liquid metal materials mainly focuses on the occurrence of a group of bubbles in solder [21,22,23,24]. Recently, research on ultrasonic cavitation has mainly focused on ultrasonic cleaning, cavitation degradation, and medical treatment. However, a cavity processing method with a controllable geometric size has not been found in metal. Ultrasonic cavitation can be divided into two states: transient cavitation and stable cavitation. Even in the stable cavitation state, the position and size of cavitation are difficult to control. This paper is based on the cavitation bubble dynamics equation [7] and standing wave position control principle [8,9]. The cavity processing method of a single cavitation bubble in low melting point alloy under the action of a composite field is studied with the aim of realizing the direct processing of a spherical cavity inside a metal droplet.

## 2. Models and Methods

The models used for acoustic cavitation and acoustic levitation vary slightly depending on their respective application fields. Typically, uniaxial acoustic field models are used, and research on acoustic cavitation and acoustic levitation is conducted separately. This paper proposes an innovative approach that combines a uniaxial standing acoustic wave field with an ultrasonic needle point acoustic field. During the experiment, these two acoustic fields are relatively independent yet coupled with each other. The standing acoustic wave field provides varying environmental acoustic pressure and acoustic radiation force, while the cavitation acoustic field provides cavitation acoustic pressure.

### 2.1. Single Acoustic Cavitations’ Model in Droplet

**Figure 1:**
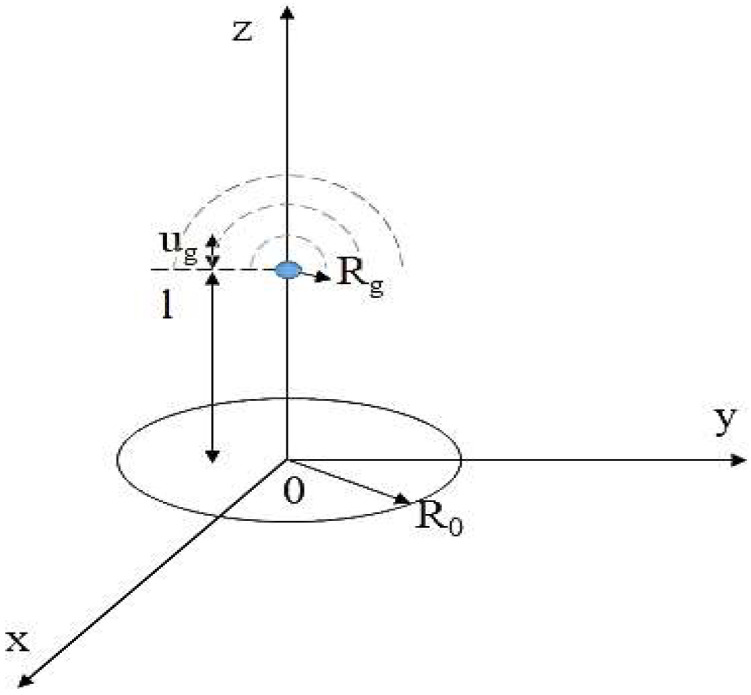
Monopole ultrasonic model at the tip of needle.

A three-dimensional coordinate system is used to establish the ultrasonic tip-monopole model, as depicted in Figure 1. The objective is to establish a theoretical research model for monopole ultrasonic cavitation.

The coordinate plane used in this model is the XOY plane with the center of the circular piezoelectric transducer as the origin. One end of the ultrasonic needle is attached to the transducer, with its vibration direction along the Z-axis direction. The local acoustic pressure model at the needle tip follows monopole vibration.

(1) $$P_{tip}=P_{g}e^{-i\left(kz-\omega t\right)},P_{g}=\rho_{g}c_{g}\omega_{g}u_{g}$$

In this equation, *u_g_* is the vibration amplitude of the needle tip, which is a constant; *ω_g_* is the angular frequency of the tip’s vibration; and *c_g_* is the acoustic propagation velocity in the Ga-In alloy. *R*_0_ represents the incident radius, *l* is the length of the needle tip, and *R_g_* is the radius of the needle tip.

The vibration of the needle tip affects the initial cavitation, causing it to be stretched and compressed by the ultrasonic vibration. The radius of the initial cavitation under the alternating acoustic pressure is described by the R-P equation. Considering the surface tension and viscosity factors, the radius variation model is as follows:

(2) $$R\ddot{R}+\frac{3}{2}{\dot{R}}^{2}=\frac{1}{\rho }P$$

*P* represents the local acoustic pressure inside and outside the cavitation bubble, and the formula is:

(3) $$P=\left(P^{\prime }-P_{v}+\frac{2\sigma }{R}\right){\left(\frac{R_{\mu }}{R}\right)}^{3\gamma }-\frac{2\sigma }{R_{\mu }}-4\mu \frac{\dot{R}}{R_{\mu }}+P_{v}-P^{\prime }+P_{tip}$$

In the initial condition at *t* = 0: *R = R_μ_*, dRdt=0, where *R_μ_* is the initial value of the bubble nucleus. In the formula, *P_tip_* is the ultrasonic pressure at the needle tip; *P’* is the environmental standing acoustic pressure; γ is the polytropic index of gas; *R* is the transient radius of the cavitation bubble; *c* is the acoustic velocity of the medium; and *σ*, *μ*, and ρ are the surface tension coefficient, viscosity coefficient, and density of the medium, respectively.

### 2.2. Compound Acoustic Fields Model of Cavitations and Levitation

**Figure 2:**
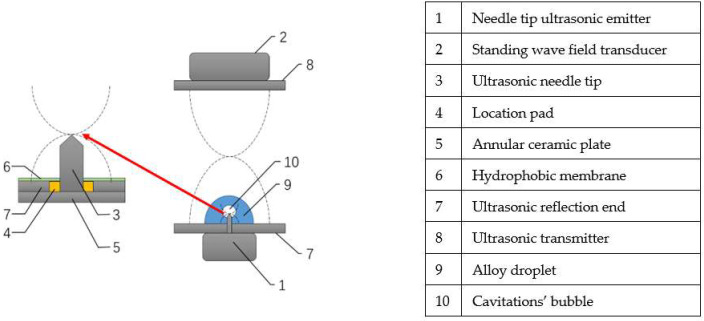
The structure and composition of the ultrasonic compound fields.

The ultrasonic cavitation and levitation composite fields model is designed by introducing the ultrasonic cavitation-levitation theory. The reflection end is transformed into the working end with a piezoelectric ultrasonic needle tip. The droplet is placed on the surface of the reflection end and the needle tip is inside the metal droplet. Its function is to realize the standing wave levitation of the droplet in the external space and the local acoustic cavitations of the needle tip inside the droplet. The structure of the ultrasonic compound fields is shown in Figure 2. The meaning of the number in the model is provided on the right side of the picture, as shown in Figure 2.

Under the action of compound acoustic fields between 7 and 8, the environmental pressure $P^{\prime }$ around the metal droplets follow the acoustic standing wave pressure law:

(4) $$P^{‘}=2P_{u}\cos\left(kz\right)\cos\left(\omega t\right)+P_{0}$$

where *P_u_* is the pressure amplitude of acoustic wave field, and *P*_0_ is the atmosphere pressure. After acoustic cavitations, Acoustic radiation force and second-order differential equation is used to describe the suspending motion of metal droplets:

(5) $$m_{p}\frac{d^{2}z}{dt^{2}}=-F-6\pi \eta ru_{rz}-\frac{1}{2}\rho_{m}v_{p}\frac{du_{rz}}{dt}-\rho_{p}v_{p}g$$

where *u_rz_* is the relative velocity of the bubble, and *m_p_* is the mass of the droplet.

Conclusively, the acoustic cavitation-levitation behaviors will follow (1)~(5) formula.

## 3. COMSOL Simulation Results and Discussion

**Figure 3:**
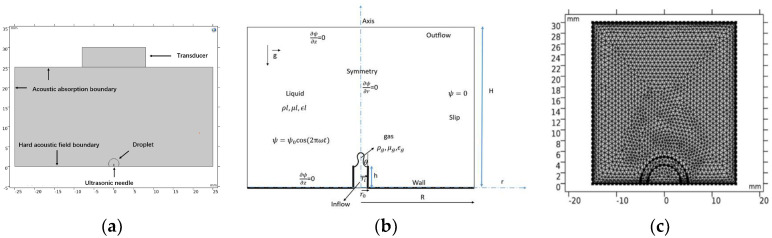
Acoustic composite fields model: (**a**) Boundary conditions of acoustic compound fields. (**b**) Computational domain for case of bubble formation from a needle, *R* = domain height, $r_{0}$ = needle outer radius, $r_{i}$ = needle inner radius, *h* = needle height, ρ = density, μ = viscosity, ϵ = relative permittivity, ψ = electric potential, ω = angular frequency of electric field oscillation, θ = contact angle with respect to needle inner surface, $\vec{g}$ is the gravity vector, subscripts *l* and *g* denote, respectively the liquid and gas phases (**c**) Grid division of composite fields model.

The setup for COMSOL simulation of acoustic compound fields involves the following steps:

(1) The acoustic cavitations are composed of the needle tip ultrasonic emitter 1, ultrasonic needle tip 3, and annular ceramic plate 5;
(2) The acoustic standing wave field in Z direction is formed by wave emission end 8 and reflection end 7;
(3) The reflection end 7 is fixed with the ultrasonic needle-tip 1, and the isolation pad 4 is used in the middle;
(4) Ga-In alloy droplet 9 was placed on annular ceramic plate 5, and the middle is isolated by a hydrophobic membrane 6. The radius of the solid–liquid interface is set as *r_g_*, and the wetting angle of the solid–liquid interface was set as *θ*. The measured droplet volume is about 1 mL;
(5) The pointer ultrasonic emitter 3 emits ultrasonic waves to the metal droplet 9 to form a focused acoustic field, which transforms cavitations into a cavity;
(6) Under the coupling effect of standing wave acoustic field and acoustic flow, the liquid surface of droplet 9 shrinks, the boundary is separated, then it is stably suspended at the standing wave node;
(7) The relationship between the length and radius of the ultrasonic needle tip in the acoustic-solid coupling fields, and the initial radius, frequency, and amplitude of the acoustic cavitation droplets were studied;

The acoustic module in COMSOL Multiphysics Software is used to visually simulate and analyze the entire computational domain, and a geometric model of the ultrasonic compound fields is established. The simulation domain is shown in Figure 3. The acoustic cavitation-levitation simulation model uses the PDE interface of the boundary partial differential equation in the form of a coefficient. For a dependent variable u:

(6) $$e_{a}\frac{\partial^{2}u}{\partial t^{2}}+d_{a}\frac{\partial u}{\partial t}+\nabla \cdot \left(-c\nabla u-\alpha u+\xi \right)+\beta \cdot \nabla u+au=f$$

Coefficients in PDE form: *e_a_* is mass coefficient, *d_a_* is damping coefficient, *c* is diffusion the coefficient, *a* is absorption coefficient, ξ is conservative flux source term, *β* is convection coefficient, and *f* is the source term corresponding to Formulas (2) and (3). Let e_a_, d_a_, and a be equal to 0:

(7) $$f=R\overset{..}{R}+\frac{3}{2}{\left(R\right)}^{2}-\frac{1}{\rho }p$$

In the CMOSOL pressure acoustic (transient state) physical field, the inner normal displacement of the transducer boundary is used to simulate the vibration of the transducer. The possible motion area around the droplet is divided into dynamic grids and the boundary probe is added to the liquid surface to calculate the surface acoustic pressure and acoustic radiation force.

In the “Two-Phase Flow, Level set (The “Two-Phase Flow” module in COMSOL Multiphysics software provides various models and methods for simulating the interaction between two or more different fluids. This includes physical phenomena, such as bubble or droplet motion, surface tension, capillary forces, and liquid turbulence in gas–liquid two-phase flow. The level set method is a mathematical approach used to describe object boundary evolution and deformation and can be applied to simulate free surface problems and interfaces between liquids and gases. In two-phase flow problems, the level set method is commonly used to track the interface between different phases. physical field, the volume force is added to the liquid phase to simulate the movement of droplets under the action of acoustic radiation force, and the displacement generated is fed back to the moving grid.

The air, Ga-In alloy, ultrasonic transducer, compound fields, piezoelectric ring, and other simulation parameters are shown in Table 1 below.

### 3.1. Local Acoustic Cavitations at the Tip Point inside Droplet

Selection of parameters: The frequency of the ultrasonic power supply is f = 30 kHz, the power is 500 W, the radius of the large end of the transducer is *D*_1_ = 58 mm, the working frequency of the piezoelectric ring PZT-8 is *ω_g_* = 15~70 kHz, and the power is 50 W. The weight of Ga-In alloy is 0.164 g, the density is 6.28 × 103 kg/m^3^, the long axis is 2a = 4.025 mm, the short axis is 2b = 2.683 mm, the aspect ratio is 1.5, and the alloy has hydrophobic contact with the reflection end. The length of ultrasonic needle is *l* = 1.2 mm, tip vibration amplitude is 3.0 μm, and the radius of needle tip is *R*_1_ = 0.15 mm. A series of air grooves with *R_μ_* = 3~12.6 μm were inscribed in the tip, and the amplitude of ultrasonic needle was 3.0 μm. The frequency solver is selected to solve the resonant frequency of the needle, and COMSOL frequency domain analysis is selected to solve the vibration characteristics of the tip.

**Figure 4:**
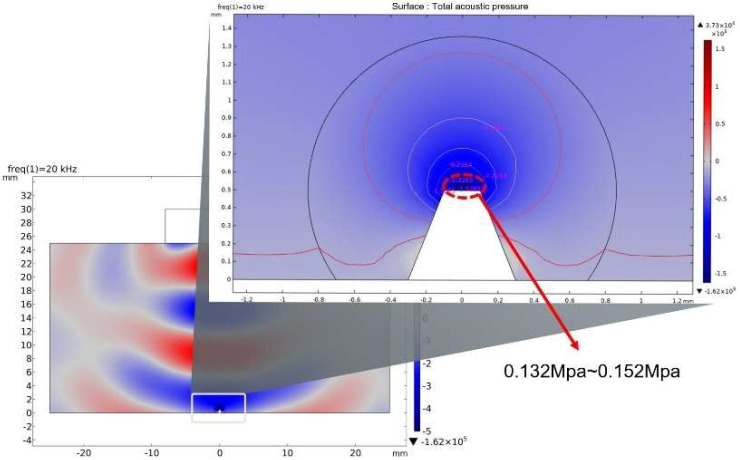
Acoustic pressure distribution of local ultrasonic cavitation field.

The condition of cavitation excitation in liquid is that the acoustics pressure should reach the cavitation threshold. Since the tip has a series of air grooves, here, if the initial radius is 12.6 μm, the theoretical threshold of acoustic cavitations of Ga-In alloy is 0.12 MPa. COMSOL simulation can intuitively show the distribution of internal ultrasonic cavitation acoustic pressure. The acoustic pressure is 0.132~0.152 MPa by adding a probe to the tip. This value exceeds the cavitation threshold and can stimulate cavitation to produce a cavity. As shown in Figure 4, under the action of piezoelectric ultrasonic needle into Ga-In alloy droplets, the local acoustic field distribution is formed inside the droplets.

### 3.2. Scale Controllability of Acoustic Cavitations’ Bubble

**Figure 5:**
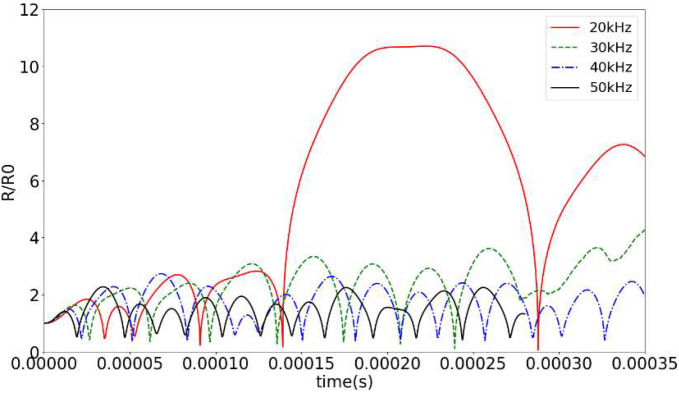
Dynamic curves of cavitations’ bubble in Ga-In alloy at different frequencies.

This study focuses on the influence of driving frequency and external acoustic pressure on cavitation intensity in previous work. Taking the initial radius of Ga-In alloy as 12.6 μm and the frequencies of 20 kHz, the dynamic behavior *R_max_*/*R*_0_ of cavitations’ bubble at different frequencies of 20 kHz, 30 kHz, 40 kHz, and 50 kHz is shown in Figure 5 below. At the driving frequency of 20 kHz, the cavitations’ bubble can expand to 11 times or more and remain stable for enough time.

**Figure 6:**
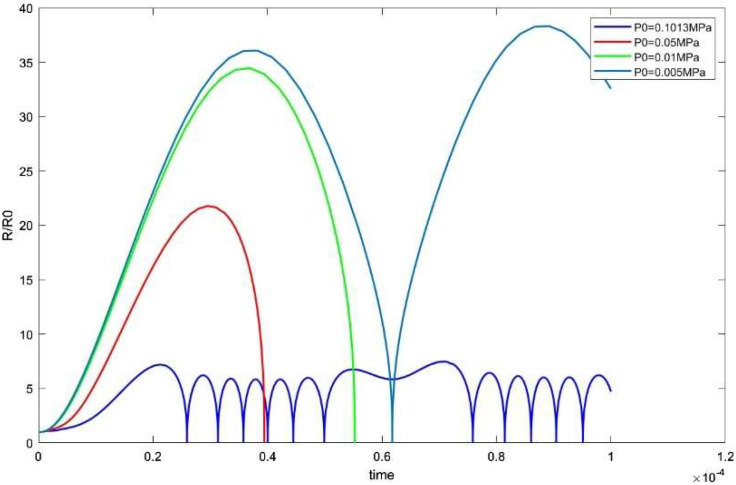
The size of cavitation bubble under different pressure $P^{\prime }$.

According to the analysis of Formulas (2) and (3) in the ultrasonic composite fields, a change in ambient pressure is more conducive to the generation of cavitation bubbles. MATLAB simulation was conducted with constant parameters and ambient pressure was reduced to 0.05 MPa, 0.01 MPa, and 0.005 MPa, respectively. The results showed that the duration and size of the cavitation bubbles were enhanced as the ambient pressure was reduced. Figure 6 illustrates this.

**Figure 7:**
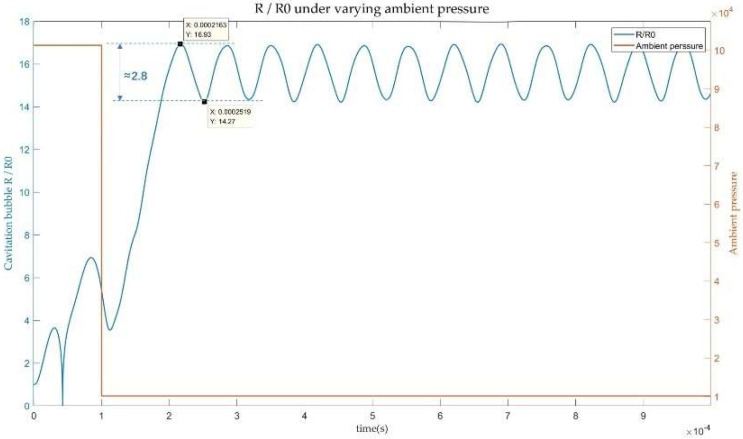
The growth curve of cavitation bubbles under the condition of changing ambient pressure.

The above analysis demonstrates that the extension of cavitation duration can be achieved by adjusting the driving frequency and reducing ambient sound pressure. To investigate the evolution of cavitation droplets in the presence of initial nuclei, while also varying the ambient sound pressure mid-process, we conducted simulation experiments. Figure 7 shows that altering the ambient pressure affects the growth characteristics of cavitation bubbles, enabling them to continue growing beyond their initial rate and maintain stable oscillations for an extended period. Its oscillation error is between 2.8 times R0. Coupling multiple variables can prolong the lifetime of cavitation bubbles and prevent their collapse, which is more effective than controlling variables separately.

### 3.3. COMSOL Simulation of Acoustic Cavitation-Levitation Process

**Figure 8:**
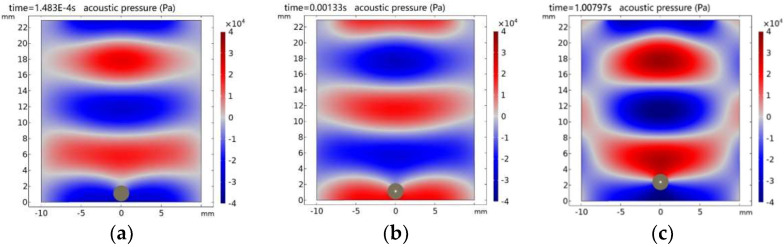
Dynamic simulation of acoustic cavitation and acoustic levitation: (**a**) Droplet and standing wave field. (**b**) Cavitations’ bubble in droplet. (**c**) Droplets suspended at standing wave node.

Based on COMSOL simulations, the acoustic levitation of droplets on the reflector involves three procedures: central shrinkage deformation, separation, and suspension. As shown in Figure 8, the time required for acoustic shrinkage deformation and separation is in the order of milliseconds, while the time required for acoustic levitation is in the order of seconds. However, the current cavitation method cannot guarantee the stable and safe inclusion of cavitation bubbles in the acoustic levitation process. Therefore, it is necessary to extend the method to coordinate with the acoustic levitation process.

### 3.4. Mechanism Analysis

**Figure 9:**
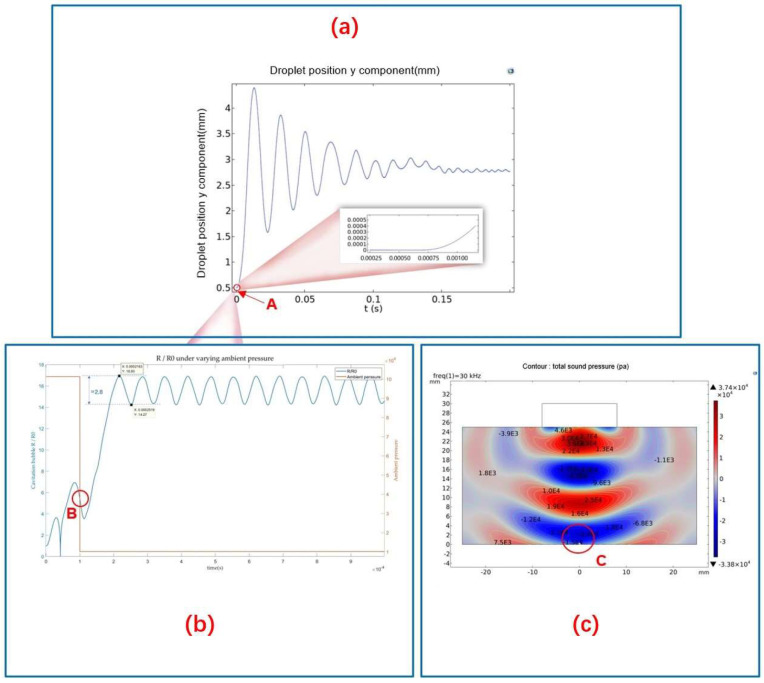
Ultrasonic composite fields cavitation bubble position and size control mechanism diagram: (**a**) Variation of liquid droplet in the y-direction during cavitation levitation process. (**b**) The growth curve of cavitation bubbles under the condition of changing ambient pressure. (**c**) Acoustic pressure distribution of stable standing wave field(the stable standing wave acoustic field provides a microgravity environment and an negative pressure for cavitation.

Using the RP equation and the theory of controlling the position of bubbles in a standing wave field, this paper proposes a new mechanism for controlling the position and size of cavitation bubbles in compound acoustics fields. Through simulation analysis, the time axis of the entire mechanism during the cavitation levitation process is explained (as shown in Figure 9a). It can be seen from Figure 9a that the time occupied by cavitation is much shorter than the levitation time during the entire process (as shown by point A in Figure 9a). Therefore, the key to the mechanism is twofold: first, to coordinate the time for entering cavitation and levitation; second, to ensure the stable size of the cavitation bubble before entering levitation. Through simulation analysis, it is found that the size of the cavitation bubble can be controlled by controlling the frequency and reducing the ambient pressure (as shown in Figure 9b for the growth curve of acoustic cavitation bubbles under a selected frequency and reduced ambient pressure). The simulation results show that compared with stable ambient pressure, the combination of different frequencies and changing ambient pressure can realize the size control of cavitation bubbles and maintain long-term stability in a negative pressure environment with an oscillation time greater than the time required to enter levitation (as shown in Figure 9b). In this process, the compound acoustics fields constructed provide a negative pressure environment (point C in Figure 9c) and provide a microgravity environment for levitation droplet, thereby achieving controllable cavitation bubble position.

## 4. Test Results

Test Contents of Ga-In Alloy Inner Cavity Fabrication are as follows:

(1) The relationship between the acoustic pressure and frequency of compound fields with the initial radius of cavitation nuclei, and the formation behaviors of acoustic cavitation bubbles are studied using Formulas (1)~(3).
(2) Cavitations are divided into transient and steady-state cavitations. The control method for the scale of acoustic cavitation cavities should be simulated and designed. The theoretical calculations and experimental conditions for controlling the scale of cavitation and critical stable suspension without bubble explosion are studied.
(3) In the acoustic compound fields, the radiation force is added to the metal droplet as a volume force to simulate its movement in the moving grids, and the displacement generated is fed back to the metal droplet with dynamic radius R of cavitation bubbles as the corresponding independent variable.
(4) Pure water droplets and low melting point Ga-In alloy are used in the tests.

**Figure 10:**
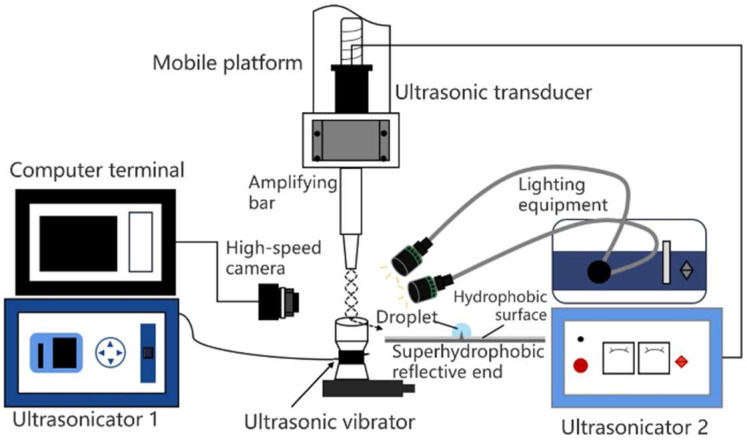
System principle of Compound acoustic fields.

The test principle in the ultrasonic droplet fabrication platform with compound fields is shown in Figure 10. The main equipment and instruments include an ultrasonic power supply, transmitter 8, reflector 7 with ultrasonic needle 1, high-speed ordinary CCD, auxiliary light source, and motion control equipment. A white light source is used to illuminate the entire acoustic field, and the shooting frequency is 10,000 frames per second, with a resolution of 1024 × 512.”

Equipment manufacturers and parameters are shown in Table 2 below:

### 4.1. Experimental Results of Acoustic Cavitation-Levitation

**Figure 11:**
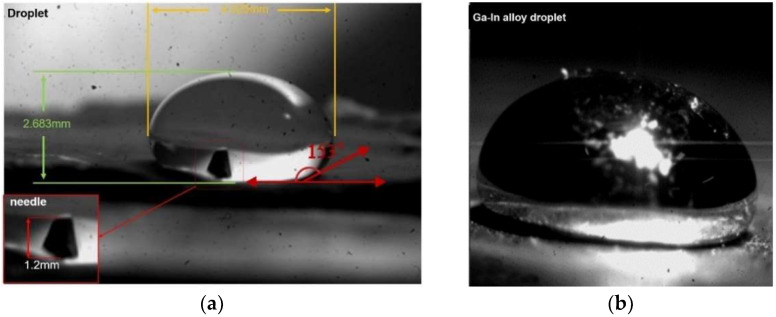
Experimental subject (**a**) Experimental water droplet and related parameters, (**b**) Experimental Ga-In alloy droplet.

The following introduces the experimental object: Since the whole experiment is a dynamic process, it is necessary to use pure water droplets as a control experiment. As shown in the following Figure 11, due to the super-hydrophobic treatment of the reflective platform, the droplets are spherical on the solid surface. The droplet is in a completely non-wetting state. The contact angle exceeds 150°, which is beneficial to the dynamic manipulation of droplets by ultrasonic composite fields.

**Figure 12:**
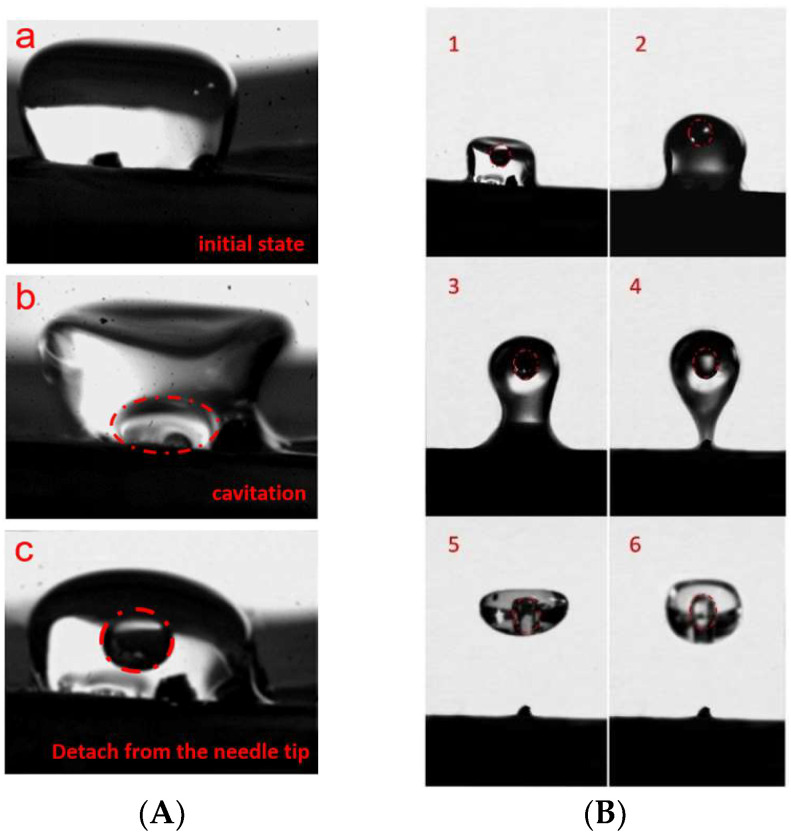
(**A**) Cavitation process of water droplets. (**B**) Droplet acoustic levitation process.

First, taking pure water as an example, the water droplet has a density of *ρ* = 1.0 g/cm^3^, surface tension coefficient of *σ* = 7.60 × 10^−2^ N/m^3^, viscosity coefficient of *μ* = 1.00 × 10^−3^ pa·s, and tip cavitation radius of *R_μ_* = 12.6 μm. Under the local ultrasonic frequency of 25 kHz, cavitation bubbles were discovered at the tip of the needle and rapidly synthesized into large radius bubbles. Pure water droplets can form cavities, and the maximum radius of cavitation bubbles can increase by approximately 17 times, as shown in Figure 12A. At the same time, the ultrasonic transmitter operates at a frequency of 30 kHz, and the height H of the compound acoustic fields is 28.82 mm. The water droplets are quickly separated from the reflection end and rise to suspend at the first standing wave node. This indicates that the method of acoustic cavitation-suspension for water droplets in the compound acoustic fields is feasible under some necessary conditions, as shown in Figure 12B.

**Figure 13:**
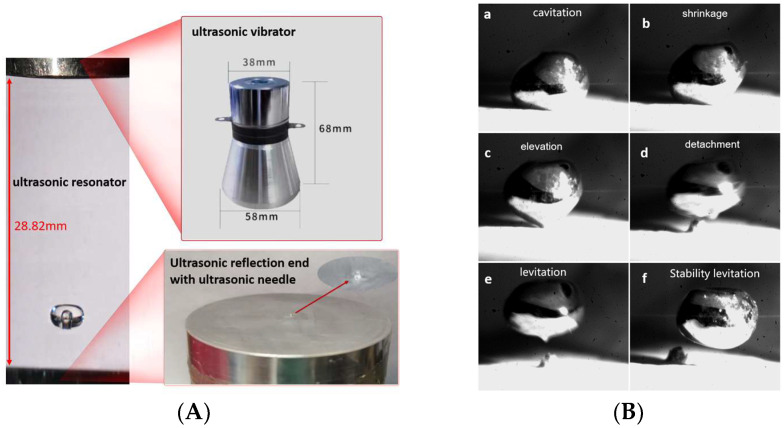
The experimental process of Ga-In droplet. (**A**) Ultrasonic composite fields experimental environment. (**B**) cavitation-levitation process of Ga-In alloy.

Second, taking Ga-In alloy droplets as an example, under the same conditions as shown in Figure 13A, with a cavitation core radius of 12.6 μm and a theoretical cavitation threshold of 0.12 MPa for Ga-In alloy, under the same tip amplitude as water droplets, the internal acoustic pressure of Ga-In alloy droplets is as high as 0.15 MPa, which exceeds the theoretical cavitation threshold. In the compound acoustic fields environment, its mass is 6.8 times that of water and its viscosity is roughly the same. Using a frequency of 25 kHz and an initial cavitation radius of 12.6 μm, the inner cavity radius of Ga-In alloy droplets is grown to 159 μm at the notch tip. During the suspending movement, the inner cavity remains stable without drifting in the compound acoustic fields environment. The whole cavitation-levitation process of Ga-In alloy mainly includes six stages, as shown in Figure 13B. The entire process was observed by a high-speed CCD.

### 4.2. Experimental Results Analysis

Under the conditions of ultrasonic composite fields, cavitation and levitation experiments were conducted on droplets with an initial bubble core size of 12.6 μm. The experimental results showed that the cavitation bubble of pure water droplets reached approximately 17 times the initial size, and the cavitation bubble radius of Ga-In alloy droplets also reached 159 μm, which was consistent with the simulation results. Additionally, after the droplet with a cavity was stably levitated in the standing wave field, the cavitation bubble remained mostly stable in the upper position of the droplet center, which was consistent with the simulated analysis position.

## 5. Conclusions

Through experiments and COMSOL simulation of whole dynamic processes, this paper clarifies the mechanism of fabricating internal microcavity in low melting point’s alloy, establishing a single and size controllable internal microcavity fabrication method by the acoustic cavitation and levitation processes in turn under compound acoustic fields. The main conclusions are as follows:

(1) The acoustic levitation of droplets includes the processes of central contraction deformation, separation, and suspension, which typically takes milliseconds to seconds. However, the acoustic cavitation process of metal droplets only takes microseconds. This study constructs compound ultrasound fields to coordinate the cavitation-levitation process, ensuring the orderly progression of cavitation and levitation processes at specific time points to generate and maintain internal cavities.
(2) Through simulation analysis, this study investigates the effects of ultrasonic frequency and ambient pressure on cavitation time and finds that reducing the frequency and negative pressure environment can prolong cavitation time. By selecting a parameter combination and coupling with the ultrasonic composite fields, the growth and stability of cavitation bubbles are successfully achieved.
(3) This study establishes an ultrasonic composite fields and clarifies the manufacturing mechanism of controllable size and position of internal cavities, which is verified by simulation and experimental analysis. The mechanism demonstrates the feasibility of internal processing within metal droplets.

As the existing laser internal processing method in transparent materials, the metal material inner cavity processing method proposed in this paper has many vigorous prospects in the inner structure’s fabrication in black materials in the future.

## Figures and Tables

**Table 1:** Calculation parameters.

Name	Parameter	Symbol	Quantity
Ultrasonic transducer	Emission end diameter Ultrasonic frequency	*D*_1_ *f*	58.0 mm 30 kHz
	Ultrasonic amplitude	*A*	15.0 μm
Acoustic compound fields	Reflector diameter	*D*_2_	300.0 mm
	Height	*H*	28.82 mm
Ga-In alloy	Melting point		289 K
	Density	*ρ_g_*	6.39 g/cm^3^
	Viscosity	*υ_g_*	0.0013 Ns/m^2^
	Surface tension	*σ_g_*	0.63 N/m
	Acoustic velocity	*c_g_*	2771 m/s
	droplet radius	*r_g_*	3.5 mm
Water	melting point		273 K
	Density	*ρ_l_*	1.0 g/cm^3^
	Viscosity	*υ_l_*	0.001 Pa/s
	Surface tension	*σ_l_*	0.076 N/m
	Acoustic velocity	*c_l_*	1481 m/s
Air	Density	*ρ*_0_	1.21 g/cm^3^
	Viscosity	*υ*_0_	0.0008937 Pa/s
	Acoustic velocity	*c*_0_	340 m/s
	Gravity acceleration	*g*_0_	9.8 m/s^2^
PZT-8 ring		*d*_33_	325 × 10^−12^ C/N
		*Q_m_*	2000
		*k*_33_	0.63
		*tanδ*	0.60%
Needle’s Tip	Materials		steel
	working frequency	*ω_g_*	15~70 kHz
	Input terminal radius	*R*_0_	4.0 mm
	Output end radius	*R*_1_	0.5 mm
	Ultrasonic amplitude		3.0 μm
	Needle tip radius	*R_g_*	0.16 mm
	Length of metal needle	*L*	1.2 mm
	Needle tip air groove	*R_μ_*	3~12.6 μm
	(Radius of cavitations’ core) Natural frequency		25 kHz
	Weber number of acoustics	*W_e_*	
	Bond number of droplet	*B_o_*	

**Table 2:** Equipment, Manufacturer, Model and Parameter Configurations.

Equipment	Manufacturer	Model	Parameter Configuration
Local ultrasonic source 1 Ultrasonic generator 2	KMD-K3-II	XT2020	Frequency: 25~45 kHz Frequency: 30 kHz
CCD	American VRI Company	MIROM310	Frequency: 10,000 frame/s Resolution: 1024 × 512
Light source Piezoelectric needle	Nanjing Yanan Special lighting	XD-300xenon	Frequency: 15~70 kHz

## Data Availability

Not applicable.

## Abbreviations

A list of symbols related to this article and its definitions are given in below:

*u_g_*	Vibration amplitude of the needle tip
*D*_1_	Emission end diameter (mm)
*D*_2_	Reflector diameter (mm)
*H*	Height (mm)
*P*	Local acoustic pressure on the cavitation bubble
*R*_0_	Input terminal radius (mm)
*R*_1_	Output end radius (mm)
*R_g_*	Needle tip radius (mm)
*L*	Length of metal needle (mm)
*R_μ_*	Needle tip air groove (μm)
*R*	Transient radius of cavitation bubble (mm)
*R_S_*	Radius of suspension droplet
*P_c_*	Cavitation threshold acoustic pressure
*P_u_*	Pressure amplitude
*ρ_m_*	Droplet and air mixing density
*ρ_g_*	Droplet mixing density (g/cm^3^)
*e_a_*	Mass coefficient
*d_a_*	Damping coefficient
*c*	Diffusion coefficient
*β*	Convection coefficient
*c_S_*	Acoustic velocity of suspension droplet
*λ_ρ_*	Volume ratio of cavity to suspension droplet
*υ_g_*	Viscosity (Ns/m^2^)
*σ_g_*	Surface tension (N/m)
*c_g_*	Acoustic velocity (m/s)
*r_g_*	droplet radius (mm)
*ρ_l_*	Density (g/cm^3^)
*υ_l_*	Viscosity (Pa/s)
*σ_l_*	Surface tension (N/m)
*c_l_*	Acoustic velocity (m/s)
*ρ*_0_	Density (g/cm^3^)
*υ*_0_	Viscosity (Pa/s)
*c*_0_	Acoustic velocity (m/s)
*g*_0_	Gravity acceleration (m/s)
*ω_g_*	working frequency (kHz)
*f*	Ultrasonic frequency (kHz)
*A*	Ultrasonic amplitude
